# Low Pretreatment Albumin-to-Globulin Ratio Predicts Poor Prognosis in Gastric Cancer: Insight From a Meta-Analysis

**DOI:** 10.3389/fonc.2020.623046

**Published:** 2021-01-26

**Authors:** Chengzhi Wei, Zhu Yu, Gonghe Wang, Yiming Zhou, Lei Tian

**Affiliations:** ^1^ Department of Emergency, The First Affiliated Hospital of Guangxi Medical University, Nanning, China; ^2^ Department of Gastroenterology Surgery, The First Affiliated Hospital of Guangxi Medical University, Nanning, China

**Keywords:** albumin-to-globulin ratio, gastric cancer, prognosis, survival, meta-analysis

## Abstract

**Background:**

In recent five years, reports regarding albumin-to-globulin ratio (AGR) and the survival of gastric cancer (GC) have emerged rapidly, yet their association remains controversial. This meta-analysis was aimed to provide an insight into the prognostic significance of pretreatment AGR in GC.

**Methods:**

PubMed, Embase, Cochrane library, Web of Science, WanFang, China National Knowledge Infrastructure (CNKI) and VIP databases were searched for relevant studies, from inception to September 30, 2020. Individual hazard ratios (HRs) with their 95% confidence intervals (CIs) were combined by Stata 12.0 software to evaluate the association between pretreatment AGR and overall survival (OS) and disease-free survival/progression-free survival (DFS/PFS).

**Results:**

A total of 8,305 patients with GC from 12 studies were included for further analysis. Pooled analyses indicated that low AGR was closely associated with worse OS (HR = 1.531, 95% CI: 1.300–1.803, *P* < 0.001) and worse DFS/PFS (HR = 2.008, 95% CI: 1.162–3.470, *P* = 0.012) in GC patients. Moreover, subgroup analyses demonstrated that the association between low AGR and worse OS remained constant despite variations in country, tumor stage, cut-off value, cut-off selection and treatment method.

**Conclusion:**

AGR could act as an efficient prognostic indicator for GC, and that low pretreatment AGR predicts poor prognosis in GC.

## Introduction

Gastric cancer (GC) is the third leading cause of cancer-related deaths globally, with 784,000 deaths in 2018 (1). Its frequently advanced stage at diagnosis leads to high mortality and poor prognosis. At present, the generally accepted prognosis indicators for GC are TNM stage, tumor differentiation and tumor location. However, patients with similar pathological features often presented diverse survival outcomes. Although the prognostic significance of certain inflammatory markers (2, 3) and tumor markers (4, 5) in GC have already been certified, we need to identify more prognostic markers that are inexpensive to test and easily available before treatment for enabling precision prediction.

Recently, the use of albumin and globulin as tumor prognostic markers have aroused great interest among scholars, due to close relations with the nutritional status and inflammatory responses of cancer patients. Albumin-to-globulin ratio (AGR) which is calculated as AGR = albumin/(total proteins−albumin) has been considered as a possible effective combination of two prognosis indicators. Previous pooled analyses indicated that lower AGR was associated with poorer survival in digestive system cancers (6), solid tumors (7), and even human cancers (8). However, regarding GC, no consensus has been reached on the role of AGR as an indicator for predicting prognosis based on the articles recently published. Thus, it is necessary to perform a meta-analysis of relevant studies to clarify whether AGR can predict the survival of GC, so as to provide more convincing evidence to confirm its prognostic value.

## Materials and Methods

### Search Strategy

A comprehensive electronic search was performed in seven databases, including four databases in English (PubMed, Embase, Cochrane library and Web of Science) and three databases in Chinese (WanFang, China National Knowledge Infrastructure (CNKI), VIP). The censor date for the present meta-analysis was up to September 30, 2020. The search terms were: (1) “albumin to globulin ratio” or “albumin to globulin” or “albumin globulin ratio” or “albumin/globulin” or “albumin and globulin” or “AGR”; (2) “gastric cancer” or “gastric neoplasm” or “stomach cancer” or “stomach neoplasm” or “cancer of stomach” or “gastric carcinoma”. No search restrictions were implemented. References from relevant literature were examined manually for potentially eligible studies.

### Inclusion and Exclusion Criteria

The criteria for eligible studies in our meta-analysis were as follows: (1) GC should be diagnosed by pathology; (2) serum albumin and globulin were measured before treatment; (3) the prognostic value of AGR for overall survival (OS), disease-free survival (DFS) or progression-free survival (PFS) in GC was explored; (4) the hazard ratio (HR) with 95% confidence interval (CI) could be extracted directly or indirectly; (5) English or Chinese articles with available full-text.

Candidate studies would be excluded according to the criteria below: (1) case reports, abstracts, reviews, comments and letters; (2) patients were not separated into high AGR and low AGR groups; (3) no sufficient data was presented to calculate HR; (4) an overlap among survival data; (5) non-human research.

### Data Extraction and Quality Assessment

Two independent inspectors (ZY and YZ) evaluated the eligibility of every candidate study by scanning the title/abstract and full-text in turn. The following information was independently extracted from the selected literature by two inspectors (ZY and GW): first author, year of publication, country, study duration, study design, sample size, tumor stage, cut-off value for AGR, cut-off selection, treatment method, follow-up time, and HR for OS/DFS/PFS with 95%CI. We designated the extraction order of HR as follows: multivariate analysis > univariate analysis > Kaplan-Meier survival curve. If only Kaplan-Meier curve could be obtained, survival data was extracted by Engauge Digitizer 4.1 to calculate HR. During the aforementioned process, disagreements between two inspectors were settled by consulting with the senior reviewers (LT).

We used Newcastle-Ottawa Scale (NOS) to score the quality of selected studies from 3 items: selection, comparability and outcome. A study with 6 stars or more was considered to be a high-quality study which was acceptable.

### Statistical Analysis

For this meta-analysis, all statistical procedures were completed using Stata 12.0 software (Stata Corp., College Station, TX, USA). The associations of low pretreatment AGR with OS and DFS/PFS were assessed by combining HRs with 95% CIs. Regarding high AGR as reference, an HR > 1 represented a negative effect of low AGR on survival outcomes. *I* ^2^ statistics were used to measure heterogeneity among studies. A random-effect model was applied when substantial heterogeneity existed (*P* < 0.1 and *I* ^2^ > 50%) (9). Otherwise, a fixed-effect model was selected. A sensitivity analysis was performed to assess whether each single study had a dramatic impact on the combined HR. Meta-regression analyses based on possible confounders were conducted to account for the heterogeneity. The publication bias was assessed from Begg’s funnel plot and Egger’s test. If a significant publication bias existed, “trim and fill method” (10) was used to adjust its potential effect. *P* < 0.05 was considered significant.

## Results

### Literature Search

**Figure 1:**
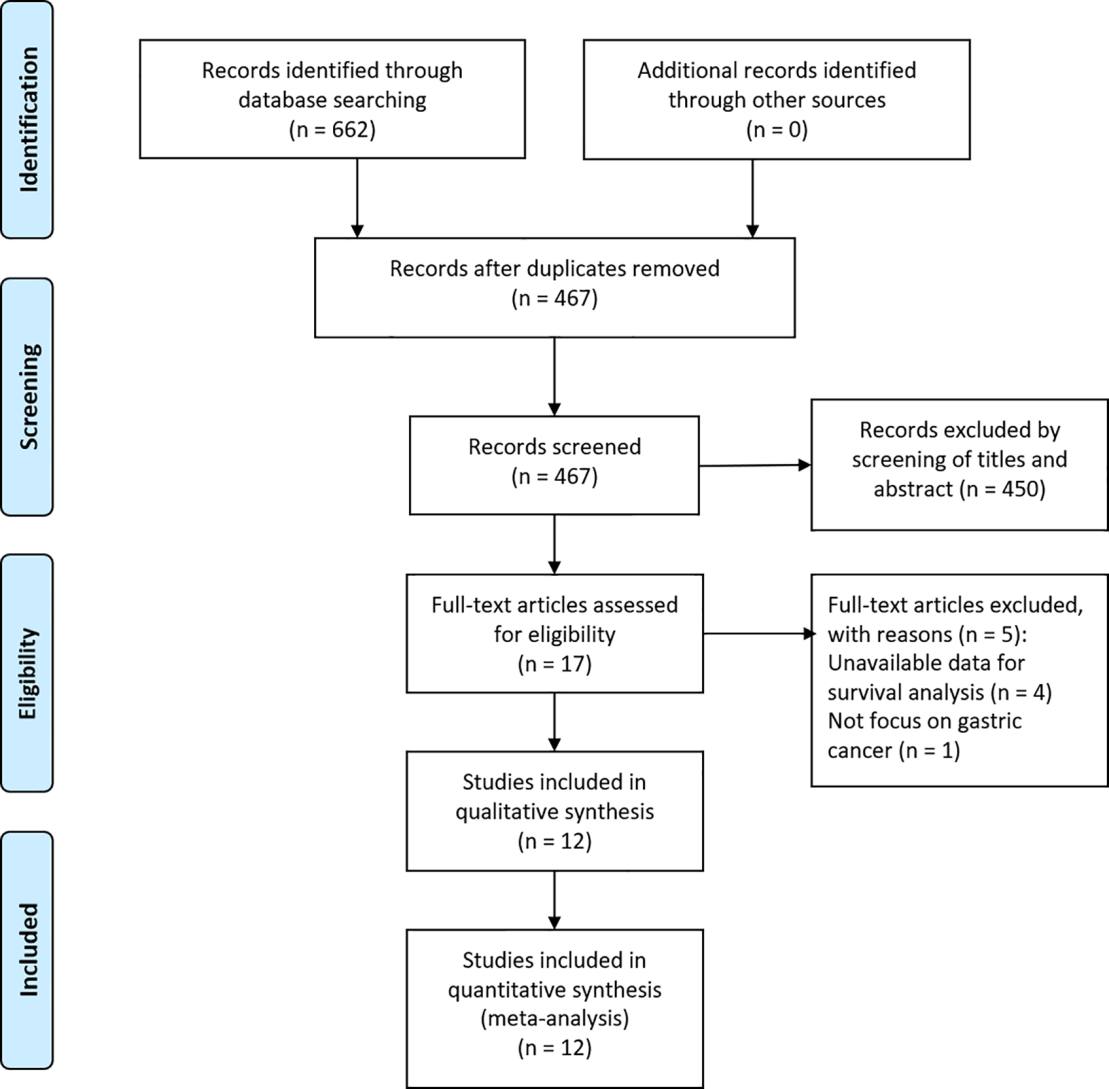
Flow diagram of the study selection.

According to the aforementioned search strategy, a total of 662 articles were yielded. After removing 195 duplicates, 467 articles were reviewed by scanning the title/abstract. Full-text analysis was performed on 17 potentially eligible studies, and 12 cohort studies (11–22) were finally applied to our comprehensive meta-analysis after excluding four with unavailable data and one that did not focus on GC (**Figure 1**).

### Study Characteristics

These 12 cohort studies, published between 2015 and 2020, included 8,305 patients with GC of TNM stage I–IV who underwent surgery and/or chemotherapy. Eleven of the 12 cohorts were retrospective, and only one (11) was prospective. The sample sizes of these cohorts ranged from 157 to 3,266. The cut-off value for AGR ranged from 1.14 to 1.93. All studies reported the association between AGR and OS, and four (11, 13, 14, 19) reported the association between AGR and DFS or AGR and PFS. Ten of studies were published in English, two (12, 16) in Chinese. The Newcastle-Ottawa Quality Score was 6–9. The summary of all cohorts was detailed in **Table 1**.

**Table 1:** The characteristics of included cohort studies.

Author	Year	Country	Study duration	Study design	Sample size	Age(years)	Tumor stage	Cut-off for AGR (high/low)	Cut-off selection	Treatment method	Follow-up (months)	Survival outcome	NOS score
Zhang et al. (11)	2020	China	2010-2014	P	273	69(median)	I-IV	1.258(171/102)	ROC	Surgery, Chemotherapy	More than 75	OS, PFS	9
Qian et al. (12)	2020	China	2012-2014	R	157	52 (mean)	I-IV	1.3(109/48)	X-tile program	Surgery, Chemotherapy	More than 20	OS	6
Xue et al. (13)	2020	China	2013-2014	R	437	65.6(mean)	I-III	1.61(NA)	ROC	Surgery, Chemotherapy	Median 63	OS, DFS	7
Bozkaya et al. (14)	2019	Turkey	2009-2016	R	251	59(median)	IV	1.2(125/126)	Median	Surgery, Chemotherapy	Median 10.2	OS, PFS	6
Xiao et al. (15)	2019	China	2008-2015	R	3266	58(median)	I-III	1.8(1417/1849)	X-tile program	Surgery	More than 60	OS	6
Han et al. (16)	2019	China	1991-2012	R	1509	60(mean)	I-IV	1.7(613/896)	NA	Surgery	Up to 120	OS	7
Mao et al. (17)	2017	China	2009-2013	R	862	58(median)	I-IV	1.5(NA)	R language	Surgery	Up to 50	OS	8
Liu et al. (18)	2017	China	2005-2012	R	507	58.8(mean)	I-III	1.93(67/440)	X-tile program	Surgery	More than 60	OS	6
Toiyama et al. (19)	2017	Japan	2001-2011	R	384	67(median)	I-III	1.3793(NA)	ROC	Surgery, Chemotherapy	Median 47.6	OS, DFS	6
Xue et al. (20)	2017	China	2007-2012	R	269	67(median)	I-III	1.36(155/114)	ROC	Surgery, Chemotherapy	Median 40	OS	6
Aksoy et al. (21)	2016	Turkey	2009-2014	R	204	NA	I-IV	1.14(153/41)	ROC	Surgery, Chemotherapy	More than 80	OS	6
Chen et al. (22)	2015	China	2007-2010	R	186	61(mean)	I-IV	1.33(NA)	X-tile program	Surgery, Chemotherapy	NA	OS	6

AGR, albumin-to-globulin ratio; DFS, disease-free survival; NA, not available; NOS, Newcastle-Ottawa Scale; OS, overall survival; P, prospective; PFS, progression-free survival; R, retrospective; ROC, receiver operating characteristic.

### The Association Between Low Albumin-to-Globulin Ratio and Overall Survival

**Figure 2:**
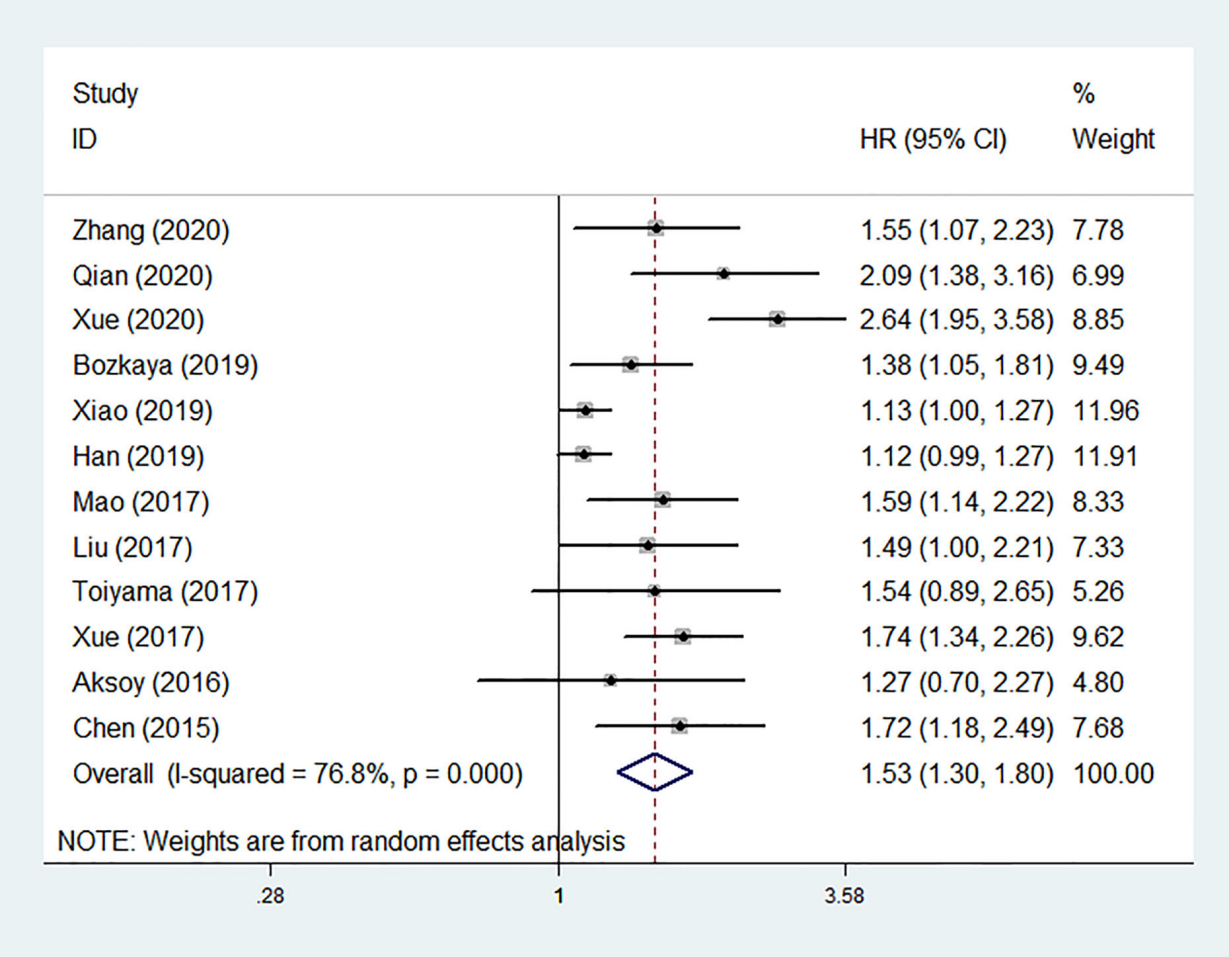
Forest plot of hazard ratios (HRs) for overall survival (OS) in total patients.

Among the 12 studies, nine reported positive results of the associations between low pretreatment AGR and worse OS, and three (16, 19, 21) showed negative results. Ten of studies provided HRs for OS in multivariate analyses, while the rest two HRs were extracted from univariate analysis and survival curve respectively (16, 20). Pooled analysis of all cohorts revealed that OS was obviously shorter in patients with low pretreatment AGR than those with elevated AGR (HR = 1.531, 95% CI: 1.300–1.803, *P* < 0.001; **Figure 2**) by using a random-effect model due to substantial heterogeneity (*I* ^2^ = 76.8%, *P* < 0.001).

**Figure 3:**
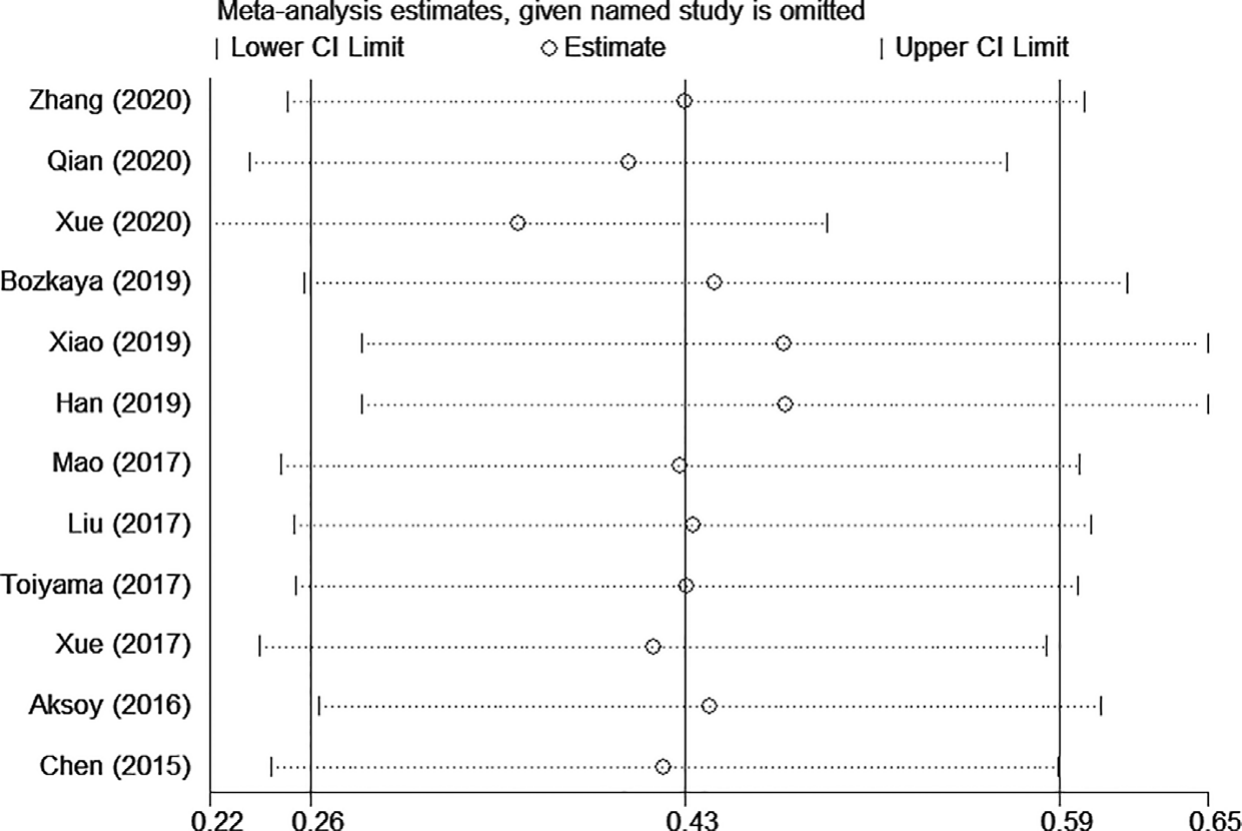
Sensitivity analysis for overall survival (OS).

**Figure 4:**
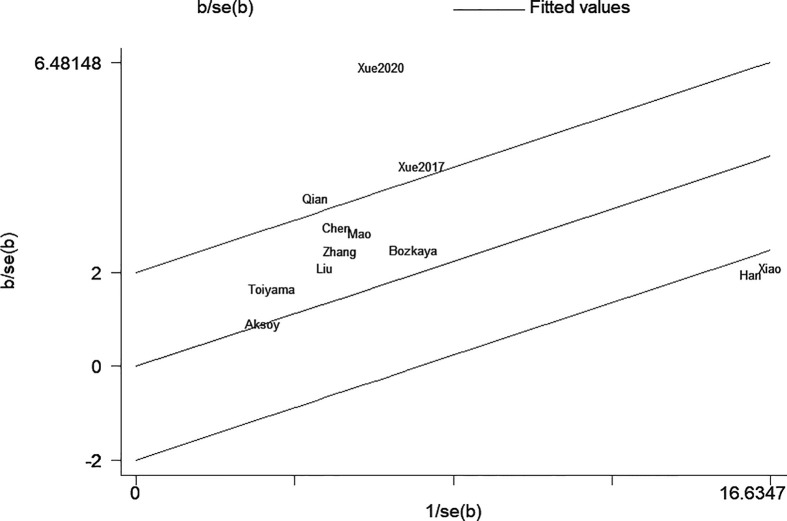
Galbraith plot for overall survival (OS).

The sensitivity analysis under a random-effect model showed that no single study dramatically affected the robustness of the pooled result across studies (**Figure 3**). Subsequently, meta-regression analyses were performed to investigate the origin of heterogeneity. We found that treatment method was a significant heterogeneous confounder (*P* = 0.022), while country, sample size, tumor stage and cut-off value were not (**Table 2**). The Galbraith plot suggested that the majority source of heterogeneity in all the selected studies was Xue’s study (13), and the other four studies presented slight heterogeneity (**Figure 4**).

**Table 2:** Meta-regression analyses for overall survival.

Variables	Coefficient	Standard error	*P*	95% CI
Country (China or non-China)	−0.120	0.211	0.583	−0.591, 0.351
Sample size (≥384 or <384)	0.100	0.165	0.557	−0.267, 0.467
Tumor stage (non-metastatic or mixed)	−0.089	0.169	0.609	−0.466, 0.287
Cut-off value (≥1.3793 or <1.3793)	0.100	0.165	0.557	−0.267, 0.467
Treatment method (surgery or multiple)	−0.337	0.125	0.022	−0.615, -0.060

CI, confidence interval.

**Figure 5:**
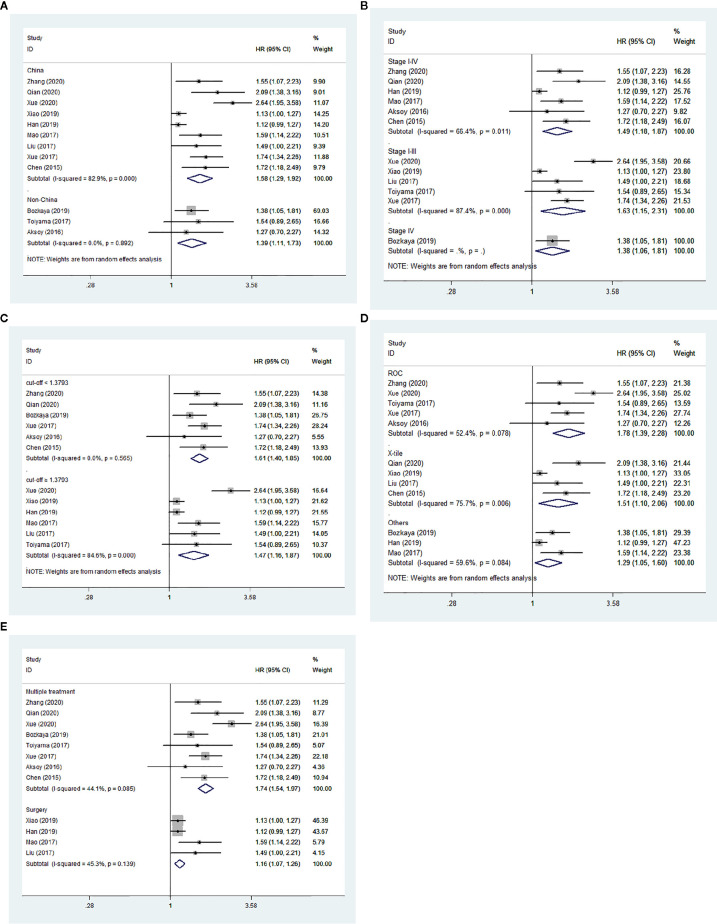
Forest plot of hazard ratios (HRs) for overall survival (OS). **(A)** Subgroup analysis stratified by country; **(B)** Subgroup analysis stratified by tumor stage; **(C)** Subgroup analysis stratified by cut-off value; **(D)** Subgroup analysis stratified by cut-off selection; **(E)** Subgroup analysis stratified by treatment method.

We performed subgroup analyses stratified by five factors (country, tumor stage, cut-off value, cut-off selection and treatment method), and the outcomes were listed in **Table 3**. Both in Chinese and non-Chinese studies, the association between low AGR and worse OS remained constant (**Figure 5A**). Similarly, we obtained consistent results in the other four subgroup analyses (**Figures 5B–E**).

**Table 3:** Subgroup analyses for overall survival.

Subgroup	No. of studies	HR (95% CI)	*P*	Heterogeneity	
				*I* ^2^ (%)	*P*
Country					
China	9	1.58 (1.29, 1.92)	<0.001	82.9	<0.001
Non-China	3	1.39 (1.11, 1.73)	0.004	0.0	0.892
Tumor stage					
Stage I-IV	6	1.49 (1.18, 1.87)	0.001	66.4	0.011
Stage I-III	5	1.63 (1.15, 2.31)	0.006	87.4	<0.001
Stage IV	1	1.38 (1.05, 1.81)	0.019	–	–
Cut-off value for AGR					
< 1.3793	6	1.61 (1.40, 1.85)	<0.001	0.0	0.565
≥ 1.3793	6	1.47 (1.16, 1.87)	0.002	84.6	<0.001
Cut-off selection					
ROC	5	1.78 (1.39, 2.28)	<0.001	52.4	0.078
X-tile program	4	1.51 (1.10, 2.06)	0.010	75.7	0.006
Others	3	1.29 (1.05, 1.60)	0.017	59.6	0.084
Treatment method					
Multiple treatment	8	1.74 (1.54, 1.97)	<0.001	44.1	0.085
Surgery	4	1.16 (1.07, 1.26)	<0.001	45.3	0.139

CI, confidence interval; HR, hazard ratio; ROC, receiver operating characteristic.

**Figure 6:**
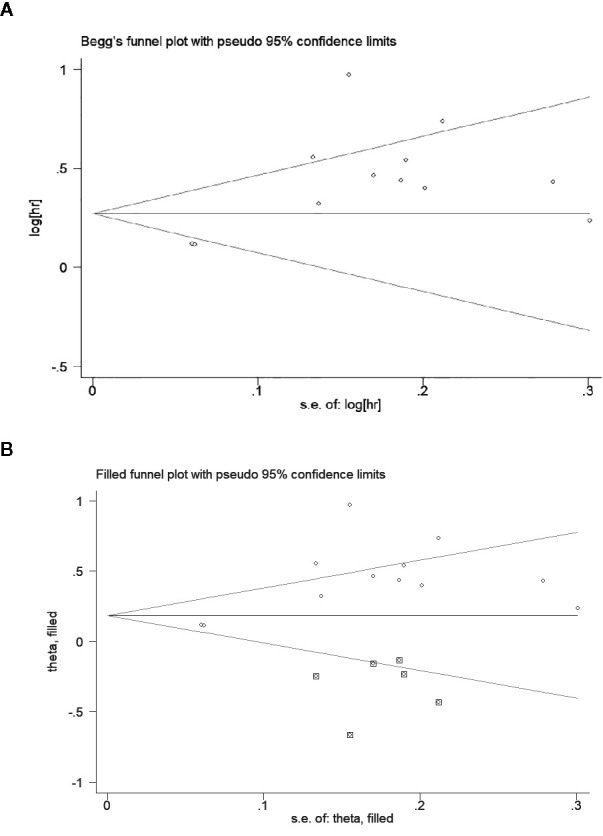
Begg’s funnel plot to evaluate potential publication bias. **(A)** Funnel plot for overall survival (OS); **(B)** The adjusted funnel plot for OS.

An obvious publication bias was observed by the asymmetric Begg’s funnel plot (**Figure 6A**) and Egger’s test (t = 3.35, *P* = 0.007). Then, we supplemented the funnel plot with 6 possible missing studies using the “trim and fill method” to make the funnel plot symmetrical (**Figure 6B**). The adjusted pooled HR under a random-effect model was 1.204 (95% CI: 1.015–1.429, *P* = 0.033), which demonstrated that the association between low AGR and worse OS was not altered after adjusting for publication bias.

### The Association Between Low Albumin-to-Globulin Ratio and Disease-Free Survival/Progression-Free Survival

**Figure 7:**
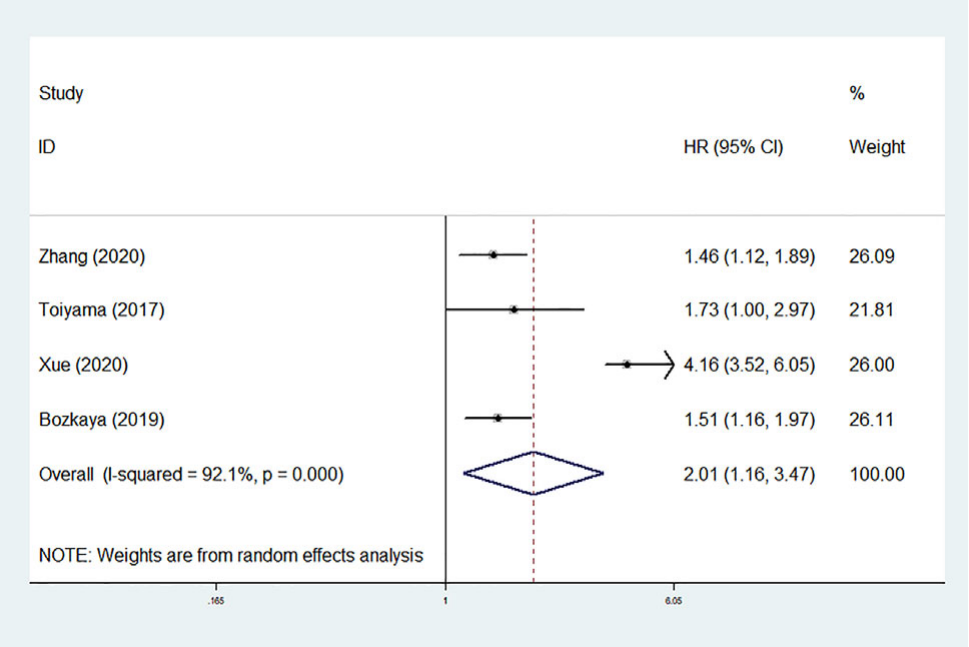
Forest plot of hazard ratios (HRs) for disease-free survival/progression-free survival (DFS/PFS).

The DFS/PFS outcomes from four studies comprising 1,345 patients were analyzed. The heterogeneity was substantial (*I* ^2^ = 92.1%, *P* < 0.001); therefore, a random-effect model was used. The result of pooling four HRs from multivariate analyses revealed that low pretreatment AGR was also significantly associated with worse DFS/PFS (HR = 2.008, 95% CI: 1.162–3.470, *P* = 0.012; **Figure 7**). Limited by the number of studies, we did not conduct test for publication bias.

## Discussion

This pooled analysis of survival data from 8,305 patients in 12 cohorts is the first large-scale study evaluating the prognostic value of pretreatment AGR in GC patients. As expected, we found that OS and DFS/PFS were obviously shorter in GC patients with low pretreatment AGR than those with elevated AGR, which indicated that low pretreatment AGR could predict poor prognosis of GC. The results in subgroups stratified by country, tumor stage, cut-off value, cut-off selection and treatment method were shown to be apparently consistent with the overall trend, which demonstrated consistent and robust effects of low AGR on worse OS. The sensitivity analysis demonstrated that the core conclusion of this meta-analysis was stable. Moreover, the reliability of core conclusion was not influenced by the appearance of publication bias.

With respect to the mechanisms of association between AGR and survival, nutrition and inflammation may be a satisfactory explanation. Patients with advanced GC are more likely to suffer from malnutrition and cachexia than those at early stages (23), which contributes to tumor progression. However, serum albumin is not only a window into the nutritional status of the human body but also a mirror of the levels of inflammation (24). Chronic inflammation has been generally accepted to be involved in the genesis and invasion of GC (25, 26). Cancer-related inflammation leads to the escape of serum albumin into the interstitium by increasing capillary permeability (27). Research has shown that albumin in the interstitium is taken up, broken down and utilized by rapidly proliferating cancer cells (28). What is more, the antioxidant function of albumin contributes to maintaining the stability of DNA replication and plays a role against carcinogenesis (29). On the other hand, the calculated globulin is thought to be a pro-inflammatory protein, including C-reactive protein (CRP), interleukin (IL), tumor necrosis factor (TNF) and so on. There is evidence suggesting that the elevation of CRP in cancer patients is caused by immune factors such as enhanced activation of macrophage function, which is closely related to revascularization of tumor and hematogenous dissemination of tumor cells (30). Moreover, the upregulation of inflammatory cytokines (such as IL-6 and TNF-α) can promote the genesis, immune escape and metastasis of GC *via* a series of pathways (31, 32), and this may also suppress the albumin synthesis (33, 34). Consequently, there is adequate biological plausibility in attributing malnutrition and inflammatory activity as the link between hypoalbuminemia and hyperglobulinemia and worse survival of GC.

Although the role of albumin and globulin alone in predicting the prognosis of GC has been confirmed (22, 35), the predictive efficacy of a single indicator is susceptible to some factors such as dehydration, fluid retention, tissue edema, synthetic raw materials insufficient, and hepatic dysfunction. However, the ratio of albumin and globulin can dramatically reduce the influence of such factors. Furthermore, the advantage of AGR also lies in its sensitivity. In previous studies (36), some subjects with both total serum protein (6.0–8.0 g/dl) and albumin (3.2–5.2 g/dl) in the normal range had low AGR (<1.1). In other words, AGR had the ability to recognize patients with poor prognosis who were not recognized by albumin. Even so, it’s worth noting that liver cirrhosis, rheumatologic diseases, as well as acute inflammation, which may cause dramatic fluctuations in protein levels, should be excluded before applying AGR to predict prognosis (37).

The value of AGR goes beyond prognostic prediction. A relatively large retrospective cohort study of a general health screened population found an increased risk of cancer incidence in subjects with low AGR, including GC (36). The study by Toiyama et al. revealed low AGR was associated with GC progression, such as large tumor size, positive lymph node metastasis, serosal invasion, and venous invasion (19). In view of the close relationships between AGR and unfavorable clinicopathologic characteristics of GC, the association between low AGR and poor survival of GC was understandable.

The high degree of heterogeneity among each study was the concern that must be taken into account. Through the sensitivity analysis, meta-regression and stratified analyses, we found that the majority of heterogeneity was attributable to treatment method. The prognostic value of AGR appeared to be higher in the multiple treatment group which included chemotherapy, possibly due to better chemotherapy tolerance in patients with good nutritional status (38). As illustrated in Galbraith plot (**Figure 4**), the heterogeneity was derived from five studies, so we conducted an in-depth analysis of these five studies. In Xue’s study (13), the subjects were non-metastatic GC patients, among whom the majority of stage II–III patients received adjuvant chemotherapy after surgery except a tiny minority in poor physical condition, which may be the reason why they obtained a high HR value. Two of the studies (15, 16) had sample sizes of more than 1,500, which were much larger than the others. Oppositely, Qian’s study (12) had the smallest sample size and the shortest follow-up time among all studies. Moreover, HR for OS in only one study (20) was calculated through the survival curve. To sum up, in addition to treatment method, the heterogeneity in our meta-analysis may also be caused by sample size, follow-up time, HR source and other factors.

Several inevitable limitations to our meta-analysis should be mentioned. First, all of the patients included in the current study were from Asian countries, so our finding about AGR may be more applicable to Asian populations. For Caucasian GC patients, the prognostic role of AGR remains unknown, but the prognostic value of pretreatment albumin has been elucidated (39). Second, the cut-off values were inconsistent which ranged from 1.14 to 1.93, hence the heterogeneity among studies may be aggravated. Third, our meta-analysis included only one prospective study, and the rest were retrospective analyses, which unavoidably led to a bias risk. Thus, further well-designed large-scale prospective trails to validate the conclusion of our meta-analysis and to explore appropriate cut-off values for different populations is indispensable.

## Conclusion

Overall, our meta-analysis demonstrated that GC patients with low pretreatment AGR compared with elevated AGR showed worse survival. Hence, we suggest that AGR could act as an efficient prognostic indicator for GC, and that low pretreatment AGR predicts poor prognosis in GC. We recommend applying AGR to identify high-risk GC patients for pretreatment intervention in clinical practice.

## Data Availability Statement

The original contributions presented in the study are included in the article/supplementary materials; further inquiries can be directed to the corresponding author.

## Author Contributions

CW and LT conceived the study and drafted the manuscript. ZY and YZ conducted the literature search. ZY and GW extracted the data. CW, GW, and YZ took part in the statistical analysis and interpreted the outcomes. CW made the figures and tables. All authors revised and checked the final manuscript. All authors contributed to the article and approved the submitted version.

## Funding

This study was supported by the National Natural Science Foundation of China (No. 81660134) and Guangxi Natural Science Foundation (No. 2017GXNSFAA198051).

## Conflict of Interest

The authors declare that the research was conducted in the absence of any commercial or financial relationships that could be construed as a potential conflict of interest.
